# Editorial for Special Issue: “Effects of Nanoparticles on Living Organisms”

**DOI:** 10.3390/cimb45020100

**Published:** 2023-02-10

**Authors:** Yoshitaka Miyamoto

**Affiliations:** 1 Department of Reproductive Biology, National Research Institute for Child Health and Development, 2-10-1 Okura, Setagaya, Tokyo 157-8535, Japan; miyamoto-ys@ncchd.go.jp or myoshi1230@gmail.com; Tel.: +81-3-3416-0181; 2 Graduate School of BASE, Tokyo University of Agriculture and Technology, Koganei, Tokyo 184-8588, Japan; 3 Department of Mechanical Engineering, Tokyo Institute of Technology, 12-2-1 Ookayama, Meguro-ku, Tokyo 152-8552, Japan

This Special Issue provides an overview of the “Effects of Nanoparticles on Living Organisms”. Nanoparticles are used in food, agriculture, drug discovery, and medicine (pharmaceuticals, prevention, and diagnosis). Most importantly, the studies reported in this Special Issue demonstrate their potential for application in the medical field, and their findings can be effectively applied to other fields. As an example of the clinical applications of nanoparticles, magnetic nanoparticles are widely used to non-invasively diagnose the structure of biological organs and tissues. Resovist^®^, a commercially available MRI contrast agent, is composed of a polysaccharide magnetic nanoparticle composite. Miyamoto et al. [1] modified the structure of Resovist^®^ to provide it with new features (cationic and anionic groups, particle size, zeta potential, etc.) and reported on the effects of nanoparticles on living organisms (3D liver models). After receiving submissions to this Special Issue and a comprehensive review process, six high-quality works were accepted for publication.

Almatroudi et al. [2] propose gene delivery via lipid nanoparticles to prevent acute inflammatory diseases. Mashima et al. [3] summarize the application of lipid nanoparticles for gene delivery and their recent clinical applications. Rewak-Soroczynska et al. [4] synthesized nanosized silicate-substituted hydroxyapatites and evaluated their cytotoxicity. Sharifi et al. [5] loaded pharmacologically active curcumin onto mesoporous silica nanoparticles and reported the effects on cancer cells. Elfaky et al. [6] studied the relationship between the size of silver nanoparticles and their hepatotoxicity. Finally, Choi et al. [7] summarize the cryopreservation of gametes and embryos using melatonin and nanoparticles.

In conclusion, we selected manuscripts that evaluate the “Effects of Nanoparticles on Living Organisms” for publication in this Special Issue, which have medical and biological applications.

Dr. Yoshitaka Miyamoto would like to thank the Editor-in-Chief, Prof. Dr. Madhav Bhatia, as well as the editorial team and the reviewers of current issues of *Molecular Biology*, who helped us in the journey towards publishing this Special Issue.

Many papers in this Special Issue report on studies in the medical field investigating the use of nanoparticles as drugs, drug delivery systems, and cryoprotectants. Therefore, we look forward to receiving submissions from authors in more diverse fields to the next Special Issue, “Effects of Nanoparticles on Living Organisms 2.0”.
